# The Influence of Design Aesthetics on Consumers’ Purchase Intention Toward Cultural and Creative Products: Evidence From the Palace Museum in China

**DOI:** 10.3389/fpsyg.2022.939403

**Published:** 2022-07-01

**Authors:** Yang Li, Jie Li

**Affiliations:** ^1^School of History, Wuhan University, Wuhan, China; ^2^Archaeological Institute for Yangtze Civilization (AIYC), Wuhan University, Wuhan, China; ^3^School of Management, Shanghai University, Shanghai, China

**Keywords:** design aesthetics, **C**hinese traditionality, perceived value, purchase intention, cultural and creative products

## Abstract

As a symbol of Chinese culture, the Palace Museum undertakes the mission of spreading Chinese culture. In recent years, through the innovative integration of traditional culture, the Palace Museum has provided a series of cultural and creative products to meet consumers’ personalized expectations, which has attracted wide attention from both academia and practice. Cultural and creative products, as one of the means of cultural dissemination by museums, affect the revenue and sustainable development of museums. Thus, it is of great significance to study how consumers arrive at the decision to purchase these products. This article explores the influence mechanism of design aesthetics on consumers’ purchase intention (*N* = 201). The results show that design aesthetics has a positive influence on consumers’ purchase intention through perceived value and that Chinese traditionality moderates the indirect influence of perceived value. The contributions and implications are discussed.

## Introduction

With the development of the economy, Chinese people are more likely to pursue quality of life and spiritual culture (Li et al., 2018). As a place that exhibits the traditional culture of past dynasties, museums naturally become a location where people can cultivate their sentiments and enrich their cultural connotations. For a long time, museums have played an important role in carrying forward excellent traditional cultures, educating the public, enlightening them, and enriching their spiritual lives (Song and Li, 2018). Furthermore, the arrival of the “Internet of Things” era has promoted the functional transformation of museums, requiring them to not only fully optimize the collection and display of cultural relics but also realize the output of cultural value through the sale of cultural and creative products. Museums need to effectively use information and communication technology to promote the spread of cultural and creative designs so that consumers can feel the cultural charm of the museum more deeply. As a representative of Chinese culture, the Palace Museum in Beijing (Forbidden City) has established the most advanced digital museum in the world with the application of information technology. The museum has vigorously launched innovative cultural and creative products and passed traditional Chinese cultural concepts on to the public through the promotion of products that have had a great influence in China and abroad (Xu, 2017).

The rapid development of the cultural and creative industry is not only meeting the spiritual and cultural needs of contemporary people but also improving the financial situation of museums themselves, which is important for their sustainable improvement. By promoting cultural products, museums can avoid financial struggles and enhance their surroundings and operating conditions (Du and Zhang, 2016), which is an inevitable way for museums to adapt to the changing world (Jiang et al., 2019). As an emerging industry, the cultural and creative industry should not only take the dissemination of traditional culture as its purpose but also consider its social and economic benefits.

If a museum intends to survive the fierce market competition, it needs to also survive severe challenges, such as resource constraints, fierce competition, and public concerns (Yu et al., 2020; Yu and Wang, 2021). In the face of these challenges, innovation is the most important factor. Product innovation is mainly reflected in design, which increases the perceived value of products and services (Li Z. et al., 2021). Consequently, innovative products win the recognition of consumers. From the perspective of design, some scholars suggest that cultural and innovative products have two functions: one is to meet people’s desires for material or non-material services, and the other is the reconstruction and interpretation of art (Nechita, 2014). Accordingly, cultural and creative products are no longer simple replications of cold history and modern utensils but need to be integrated into modern social life. The design of cultural and creative products should fit modern life and pay attention to the integration of modern science and technology (Nechita, 2014) in order to firmly grasp the attention of the audience. With its special design, the Palace Museum has steadily gained the admiration of people through the integration of Chinese cultural elements and internet technology, digital technology and new media technology. Also, online and offline customer services and a cultural exchange space need to be built to create a new user experience so people can become more familiar with the Forbidden City and a better inheritance and traditional Chinese culture dissemination can be promoted (Nechita, 2014).

The overwhelming acceptance of cultural and creative products by the public is reflected in their sales volume. Therefore, it is of great importance to study consumers’ intention to purchase cultural and creative products, which affects whether traditional culture can be transmitted in a lasting and far-reaching manner. Consumer purchase intention is the basis of consumer purchase behavior (Ajzen, 2002), and it refers to the fundamental cognitive psychology and content material to the patronization of products or services, considering the stimulation of exterior factors, and is an essential indicator that can predict patron shopping for conduct (Gill et al., 2005). Research on purchase intention is mainly reflected in its influencing factors, such as individuals’ identities (Chetioui et al., 2020), the atmospheres of social media platforms (McClure and Seock, 2020), advertisement information (Alalwan, 2018), brand awareness (Shahid et al., 2017), and corporate reputation (Bianchi et al., 2019). Although scholars have conducted many studies on purchase intention, the purchase intention of the Forbidden City cultural and creative products is still limited, and no in-depth inquiry has been carried out. Therefore, this study commences by outlining the elements affecting consumers’ purchase intention of cultural and creative products and discusses the influence mechanism of design aesthetics on consumers’ purchase intention. In addition, perceived value and Chinese traditionality are introduced as the mediating and moderating variables respectively, to further explore the influencing mechanism with the aim of providing guidance for the further promotion of museums’ cultural and creative products.

## Hypothesis Development

### Design Aesthetics and Purchase Intention

In behavioral research, the concept of aesthetics was first proposed by Fechner (Liu, 2003). Its purpose is to find the relationship between different design dimensions and perceived attraction by the design of rectangles and ellipses with visual impact, to determine the factors affecting the attraction of objects, such as shape, tone, color and texture (Liu, 2003). With the deepening of research on aesthetics, scholars have begun to pay attention to design aesthetics. Toufani et al. (2017) describes design aesthetics as a sensory perception of beauty, order and harmony; Schultz (2005) believes that design aesthetics is a kind of art, balance or emotional appeal that can be expressed through elements such as color, shape, and music. Moreover, design aesthetics is also considered a communication tool to interpret product functions; tangible and visual design features are carriers to convey information such as aesthetics, function, and symbolic significance of innovative products to consumers (Talke et al., 2009).

Compared with business products, the cultural and innovative products of museums are viewed more as carriers of cultural expression rather than commodities. Therefore, when they are sold, consumers’ doubts are easily aroused, and this may weaken consumers’ purchase intention and hinder the development of cultural and creative industries. However, existing studies have pointed out that design aesthetics can significantly affect consumer behavior (Cai and Xu, 2011; Hagtvedt and Patrick, 2014), and that product design aesthetics can influence consumers’ first impression (Diefenbach and Hassenzahl, 2011). When product design draws the interests or emotions of consumers, they may not care too much about the price of the product (Norman, 2004). Additionally, some research has pointed out that there are enormous variations in consumers’ purchase intentions for products with high design aesthetics and products with low design aesthetics (Shi et al., 2021). Therefore, design aesthetics has a positive influence on consumers’ purchase intention (Hagtvedt and Patrick, 2014). Based on this, we propose the following:

**H1:** Design aesthetics is positively correlated with consumers’ purchase intention.

### The Mediating Role of Perceived Value

The notion of perceived value was first presented in Porter’s competitive advantage (Porter, 1985). Thereafter, scholars have proposed definitions of perceived value based on different perspectives. Zeithaml (1988) opined that perceived value can be regarded as consumers’ overall evaluation of product utility, as nicely as consumers’ perceived preference and evaluation of product features and attributes (Woodruff, 1997). Moreover, scholars have divided perceived value into dimensions, and some scholars believe that a product’s perceived value has intangible and tangible dimensions (Snoj et al., 2004). Sweeney and Soutar (2001) put forward four dimensions of consumers’ perceived value: quality value, functional value, social value, and emotional value. Sheth et al. (1991) believed that perceived value could be divided into five value dimensions—namely, functionality, emotion, sociality, cognition, and conditionality—and pointed out that all these would affect consumers’ purchasing decisions.

In this article, we endorse that perceived value performs a mediating function in the relationship between design aesthetics and consumers’ purchase intention. Existing literature suggests that although cultural and creative products can also be questioned by consumers when they are sold as commodities, design aesthetics can alleviate such feelings. On the one hand, design aesthetics can improve product functions (Hagtvedt and Patrick, 2014), which can promote consumer trust in the product and develop the perceived value of the product. On the other hand, design aesthetics can increase the potential value of products and influence consumers’ preferences (Stanton et al., 2016). The response of consumers to products with high design aesthetics has a positive impact on their judgment of products—that is, design aesthetics has a positive impact on consumers’ perceived value (Schultz, 2005). Therefore, cultural and creative products with high design aesthetics may be more likely to trigger consumers’ perceived value than those with low design aesthetics.

In addition, based on the analysis of utility theory, perceived value is a trade-off between the benefits obtained from the transaction and the costs paid by consumers. No matter the consumption situation, the purchase purpose of consumers is to obtain maximum utility at the minimum cost. Therefore, under the condition of their own limited resources, consumers will first decide whether to buy goods based on their subconscious judgment of the utility of the goods. The higher the value of goods as perceived by consumers, the more willing they are to pay (Zeithaml, 1988). Eggert and Ulaga (2002) also found that consumers make purchase decisions in accordance with the maximization of perceived value, believing that perceived value is the most important factor that truly drives users to pay. Kleijnen et al. (2007) and Ko et al. (2009) also found that consumers’ perceived value of products and services can well explain their purchase intention when studying consumers’ purchasing behaviors. Therefore, consumers’ perceived value will affect their purchase intention (Wood and Scheer, 1996; Al-Sabbahy et al., 2004; Chen and Chang, 2012; Weng and de Run, 2013). Based on this, we propose the following:

**H2:** Perceived value mediates the relationship between design aesthetics and purchase intention.

### The Moderating Role of Chinese Traditionality

Chinese traditionality is a cultural variable that describes the fundamental cognition, concept, attitude, conduct and values below the impact of regular culture, and refers to the degree of personal identification with traditional Chinese values (Hui et al., 2004). Chinese traditionality comprises five dimensions—namely, obedience to authority, filial piety and ancestor worship, conservatism and patience, fatalism and defense, and male dominance (Yang et al., 1989). Among them, obedience to authority is the most outstanding dimension (Farh et al., 1997, 2007). In organizations that center attention on obedience to authority, Chinese traditionality is described as the degree to which humans apprehend and adhere to the typical hierarchical position of relationships in Confucian social ethics (Farh et al., 1997).

Based on the theory of cultural self-representation, this article explores the mechanism of moderated mediation. The theory holds that there is a connection between the macro level of culture and the micro level of individual behavior, and people’s behaviors are influenced *via* the formation of cognition through the interpretation of culture (Erez and Earley, 1993). More specifically, the information in a cultural environment is endowed with meaning by individuals and influences individual behavior through the process of selective identification, evaluation, and interpretation (Erez and Earley, 1993). There is a large amount of literature that introduces Chinese traditionality into the study of individual behavior, focusing on its influence on individual perception, motivation, attitude, and behavior (Kirkman and Shapiro, 2001; Wang et al., 2002; Ng et al., 2009; Zhao et al., 2019). Studies have found that individual behavior may be influenced by Chinese traditionality (Farh et al., 2007; Zhang et al., 2014), as individuals with high levels of Chinese traditionality may be more inclined to produce certain behaviors (Hui et al., 2007; Ngo and Li, 2015; Li J. et al., 2021). Thus, we believe that Chinese tradition, as an important factor affecting individual cognition and behavior (Farh et al., 1997, 2007; Hui et al., 2004; Spreitzer et al., 2005; Chen and Aryee, 2007), may also have a certain impact on consumers’ purchasing behavior.

The theory of rational behavior points out that behavioral intention drives rational behavior and is influenced by attitudes related to behavior (Douglass, 1977; Karnowski et al., 2018). Also, attitudes depend on individual beliefs about object attributes or behavioral outcomes (Montano and Kasprzyk, 2015)—for example, people who believe that a behavior will produce a positive value will hold a positive attitude toward the behavior (Bang et al., 2000). In this article, high Chinese traditionality means that individuals perceive cultural and innovative products with design aesthetics as having a higher symbolic value of traditional Chinese culture and can better spread Chinese culture. In this case, consumers will have a positive attitude toward cultural and creative products, which enhances their perceived value of these products, and this consequently generates willingness and action to purchase them.

However, low Chinese traditionality means that the level of individuals’ perception of traditional Chinese culture symbolized by the cultural and creative products of museums is low, which will have a negative impact on the dissemination of Chinese culture. According to rational action theory, people who perceive negative results will stimulate their negative attitudes (Bang et al., 2000), so consumers who think the cultural and creative products of museums have low symbols of traditional Chinese culture will have a negative attitude toward them, which is not conducive to consumers’ perceived value of cultural and creative products. Hence, we propose that Chinese traditionality level moderates the relationship between design aesthetics and perceived value.

**H3:** Chinese traditionality moderates the relationship between design aesthetics and perceived value, and this relationship is stronger when Chinese traditionality is high rather than low.

Based on the assumption of H1 and H3, we propose a moderated mediation model—that is, Chinese traditionality moderates the indirect effect of design aesthetics on purchase intention *via* perceived value. Therefore, we assume the following:

**H4:** Chinese traditionality moderates the indirect effect of design aesthetics on purchase intention *via* perceived value, such that the indirect effect is stronger when perceived value is high rather than low.

**FIGURE 1:**
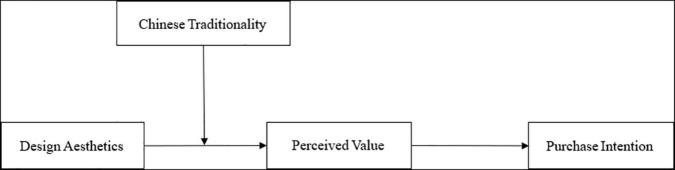
Hypothesized conceptual model.

Figure 1 shows the theoretical model used in this article.

## Materials and Methods

### Sample and Procedures

The data in this article were collected in a university town in eastern China, and the participants were students in this area. We used the snowball sampling procedure to collect the data (Goodman, 1961) by informing them of the academic research purpose of the questionnaire and the confidentiality of the questionnaire information, thus ensuring the authenticity of the data. In total, 201 valid questionnaires were collected. Among them, 65.67% were men, 34.32% were women. Participants answered questions on a five-point Likert scale (1 = completely disagree to 5 = strongly agree).

### Measures

#### Design Aesthetics

Design aesthetics was measured with a scale from Hsiao (2013), which consisted of three items and adopted a five-point Likert scale (ranging from 1 = completely disagree to 5 = strongly agree). Participants were asked to indicate the extent to which they agreed with the design aesthetics items. A sample item was “I am satisfied with the design of the product.” Cronbach’s alpha was 0.93.

#### Perceived Value

Consumer perceived value was measured with a scale from Gounaris (2005), which consisted of four items and used a five-point Likert scale (ranging from 1 = completely disagree to 5 = strongly agree). Participants were asked to indicate the extent to which they agreed with the perceived value items. Cronbach’s alpha was 0.86.

#### Chinese Traditionality

Chinese traditionality was measured with a scale from Farh et al. (2007), which consisted of four items and used a five-point Likert scale (ranging from 1 = completely disagree to 5 = strongly agree). Participants were asked to indicate the extent to which they agreed with the Chinese traditionality items. Cronbach’s alpha was 0.85.

#### Purchase Intention

We adopted the scale by Lu and Hsiao (2010), which consisted of three items and used a five-point Likert scale (1 = completely disagree to 5 = strongly agree). Participants were requested to indicate the extent to which they agreed with the purchase intention items. Cronbach’s alpha was 0.89.

#### Control Variables

According to the existing research on consumers’ purchase intention, gender, age, and education level were selected as control variables to exclude the potential influences of exogenous variables.

## Results

### Confirmatory Factor Analysis

Confirmatory factor analysis was performed using structural equation modeling (Amos 25.0), and the data fit indexes of the four-factor model met the fitting requirements (χ^2^/*df* = 2.02, RMSEA = 0.07, IFI = 0.96, CFI = 0.96, TLI = 0.95), indicating good discriminative validity among the variables. The details are shown in Table 1.

**TABLE 1:** Results of confirmatory factor analysis.

Model	Contained factors	χ^2^	*df*	χ^2^/*df*	RMSEA	CFI	IFI	TLI
Single factor model	PI + CI + DA + PV	902.57	77	11.72	0.23	0.54	0.54	0.45
Two-factor model	PI + CI; DA + PV	794.19	76	10.45	0.22	0.60	0.60	0.52
Three-factor model	PI; CI; DA + PV	532.47	74	7.20	0.18	0.74	0.75	0.68
Four-factor model	PI; CI; DA; PV	143.32	71	2.02	0.07	0.96	0.96	0.95

*N = 201.*

*PI, purchase intention; CI, Chinese traditionality; DA, design aesthetics; PV, perceived value.*

### Descriptive Statistics and Correlations

Table 2 presents the descriptive statistics and correlations among the main variables. As displayed, design aesthetics is positively correlated with purchase intention (*r* = 0.43, *p* < 0.01), which adequately supports Hypothesis 1. Additionally, design aesthetics also has a positive relationship with perceived value (*r* = 0.26, *p* < 0.01), and perceived value is significantly correlated with purchase intention (*r* = 0.48, *p* < 0.01). Moreover, Chinese traditionality is positively related to design aesthetics (*r* = 0.57, *p* < 0.01), perceived value (*r* = 0.46, *p* < 0.01), and purchase intention (*r* = 0.41, *p* < 0.01).

**TABLE 2:** The means, standard deviations, and correlations among the study variables.

	Mean	SD	1	2	3	4	5	6	7
1. Design aesthetics	4.71	1.36	–						
2. Perceived value	5.19	1.07	0.26**	–					
3. Chinese traditionality	4.38	1.30	0.57**	0.46**	–				
4. Purchase intention	4.98	1.31	0.43**	0.48**	0.41**	–			
5. Gender	1.34	0.48	0.05	−0.06	0.04	−0.06	–		
6. Education	2.53	0.76	−0.09	−0.07	−0.09	−0.17*	−0.07	–	
7. Age	25.83	2.48	−0.03	−0.00	0.04	−0.00	−0.06	0.22**	–

*N = 201. Gender: 1 = male, 2 = female.*

*p < 0.05; **p < 0.01.

### Hypothesis Testing

As shown in Table 3, we used a series of multiple regression analyses to examine our hypotheses. We started by examining the direct effects, followed by the mediating effect and finally analyzed the moderating effect. Model 1 examined the influence of the control variables on purchase intention. Model 2 added design aesthetics to test its main influence on purchase intention. To clarify the mediating effect, Model 4 added perceived value and design aesthetics to test their influence on purchase intention.

**TABLE 3:** Results of the moderated regression analyses.

Variables	Purchase intention				Perceived value			
	Model 1	Model 2	Model 3	Model 4	Model 5	Model 6	Model 7	Model 8
**Control variables**								
Age	0.03	0.04	0.03	0.03	0.01	0.01	−0.02	−0.05
Gender	−0.07	−0.08	−0.04	−0.06	−0.07	−0.08	−0.08	−0.06
Education	−0.18*	−0.15*	−0.14*	−0.12*	−0.08	−0.06	−0.03	−0.01
**Independent variable**								
Design aesthetics		0.42***		0.32***		0.26***	0.00	0.05
**Mediator**								
Perceived value			0.47***	0.39***				
**Moderator**								
Chinese traditionality							0.46***	0.47***
**Interaction term**								
Design aesthetics × Chinese traditionality								0.25***
*R* ^2^	0.03	0.21	0.25	0.35	0.01	0.08	0.22	0.27
*ΔR* ^2^	0.03	0.18	0.22	0.14	0.01	0.07	0.14	0.06
*F*	2.26	13.00***	16.58***	20.83***	0.62	4.06**	10.85*****	12.21***

*N = 201. Design aesthetics, Chinese traditionality, and their interaction were centered prior to analysis.*

*p < 0.05; **p < 0.01; ***p < 0.001.

As shown in Table 3, design aesthetics was positively correlated with purchase intention (β = 0.42, *p* < 0.001, Model 2), which supports Hypothesis 1. Model 4 added mediation variables based on Model 2, and the results of Model 4 displayed a significant mediating effect of perceived value on the relationship between design aesthetics and purchase intention (Δ*R*^2^ = 0.35, *F* = 20.83, *p* < 0.001). Although it was decreased for the regression coefficient of design aesthetics on consumers’ purchase intention (β decreased from 0.42 to 0.32, *p* < 0.001), perceived value still had a significant impact on purchase intention (β = 0.39, *p* < 0.001). That is, perceived value partially mediates the influence of design aesthetics on purchase intention. Thus, Hypothesis 2 is supported.

**FIGURE 2:**
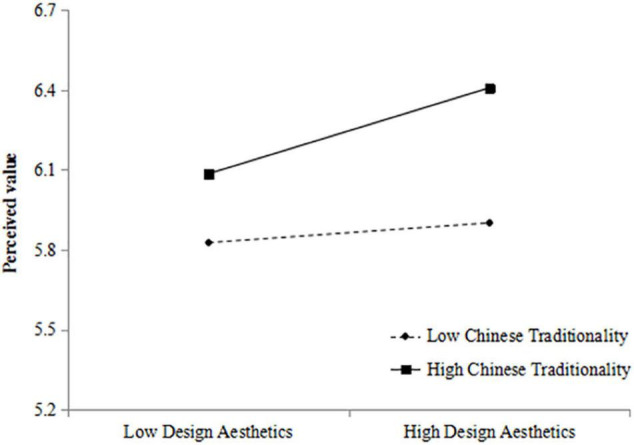
Interaction of design aesthetics and Chinese traditionality on perceived value.

Table 3 shows the hierarchical regression results of Chinese traditionality of the relationship between design aesthetics and perceived value. At the same time, the data variables were centralized to reduce the collinearity problem in the regression process. The product term “design aesthetics × Chinese tradition” was significantly related to perceived value (Model 8: β = 0.25, *p* < 0.001), proving the moderating effect of Chinese traditionality and verifying the inference of Hypothesis 4. Figure 2 shows the pattern of this interaction. In this article, high Chinese traditionality and low Chinese traditionality were obtained by adding and subtracting one standard deviation from the mean of Chinese traditionality. As shown in Figure 2, compared with low Chinese traditionality, the higher the Chinese traditionality, the stronger the positive relationship between design aesthetics and perceived value. Therefore, Hypothesis 3 is supported by the data.

In this study, the SPSS PROCESS macro was used to test the mediating effect of Chinese traditionality. According to the results obtained, in the context of low Chinese traditionality, the indirect effect of perceived value through the relationship among design aesthetics and purchase intention was non-significant (95% confidence interval [−0.1393, 0.0314]). In the context of high Chinese traditionality, perceived value had a significant mediating effect between design aesthetics and purchase intention (95% confidence interval [0.0226, 0.1986]). This suggests that the mediating effect of perceived value is affected by different levels of Chinese traditionality. That is, the higher Chinese traditionality is, the stronger the mediating effect of perceived value. In addition, according to the index of moderated mediation, the indirect effect of perceived value between design aesthetics and purchase intention was significant (95% confidence interval [0.0219, 0.0984]). Therefore, Hypothesis 4 is supported.

## Discussion

Based on Chinese cultural background, this study concentrates on the cultural and creative products in the Palace Museum, which respond to the appeal of promoting traditional Chinese culture and provide a new way to improve consumers’ purchasing intention of cultural and creative products. Specifically, our study explores how design aesthetics affects consumers’ purchase intention of cultural and creative products through perceived value. The results show that design aesthetics has a positive influence on purchase intention; perceived value plays a mediating role between design aesthetics and purchase intention; Chinese traditionality not only moderates the relationship between design aesthetics and perceived value but also significantly moderates the mediating role of perceived value between design aesthetics and purchase intention. Specifically, the higher the level of Chinese traditionality in a consumer, the stronger the influence of design aesthetics on purchase intention *via* perceived value and vice versa.

### Theoretical Implications

This research has the following theoretical significance: First, design aesthetics is introduced into the cultural and creative industry to fill the gaps in previous studies and expand the theoretical research of design aesthetics. Existing studies on design aesthetics mainly focus on the field of fine arts and design, and few studies apply design aesthetics to the cultural industry. This study explores the influence mechanism of design aesthetics on consumers’ purchase intention, which is innovative to some extent.

Second, it enriches and deepens theoretical research on Chinese traditionality. The previous research on the Chinese tradition typically focuses on management and explores the influence of Chinese traditionality on leadership style and employee performance. This study, however, introduces Chinese traditionality into the field of cultural entrepreneurship, explores the moderating role of Chinese traditionality in the consumer decision-making process—that is, when considering factors affecting consumers’ purchase intention, it is necessary to consider both product characteristics and cultural attributes.

### Practical Implications

This study aims to explore how design aesthetics affects consumers’ purchase intention of cultural and creative products of the Palace Museum, and the research results can provide some practical enlightenment for practitioners and designers of cultural and creative products.

Initially, product managers should be deeply aware that consumers focus on experiential consumption and are becoming increasingly fond of innovative products. Thus, product managers ought to pay more attention to the design aesthetics of cultural and innovative products, integrate Chinese traditionality into cultural and creative products and ensure that the products are innovative and unique. They can bring in interdisciplinary talents who no longer solely understand the history and culture of the museum collection but also have additional knowledge about the development and design of cultural and innovative products and can improve product characteristics, thereby meeting the needs of consumers.

Moreover, when designing cultural and innovative products, designers should pay attention to the perceived value of the products, as it is only when consumers feel the products are valuable that they will purchase them. The following specific measures can be taken. The first is to combine culture with virtual reality technology to develop digital cultural and creative products with personalized experiences. Second, museums can launch DIY projects online and offline according to their collections, so consumers can participate in the production process of cultural and innovative products and further understand the value that the products represent. Third, museums can cooperate with colleges to set up behavior experiential courses such as craft making, situation simulation, and so on. Participants can acquire historic and cultural knowledge *via* such immersive and interactive courses, and the perceived value of products can be enhanced. This will lead to the purchase of more products and the realization of the sustainable development of museums.

Finally, practitioners should strike a balance between innovation and the seriousness of the Chinese cultural tradition. Instead of excessively imitating traditional culture to meet the demand of consumers for personalized experience, managers should pay attention to Chinese tradition and develop designs and products based on historical facts. The cultural and innovative industry must and can only serve as “the icing on the cake” of museums. The original intention of spreading the traditional Chinese culture should be adhered to so that consumers can learn traditional Chinese culture in the process of happy shopping.

### Limitations and Future Research

Although this article supplements the research on consumers’ purchasing intention with some innovation and insights, it also has some limitations. First, the participants selected for this study are concentrated in some universities in eastern China. Although representative to a certain extent, the sample size may not be large enough, which may affect the universality of the results. As such, future studies could further expand the sample size. Second, this study focused on purchase intention rather than actual purchase behavior. Yuan et al. (2019) found a high correlation between customer intention and actual usage, but intention did not always lead to actual behavior. Therefore, future research can explore actual purchase behavior beyond purchase intention. Lastly, our sample solely included a unique human group in China, and whether the findings can be utilized for different cultural groups remains to be explored. Future research could explore the impact of cross-cultural research to complement our findings.

## Data Availability Statement

The raw data supporting the conclusions of this article will be made available by the authors, without undue reservation.

## Ethics Statement

Ethical review and approval was not required for the study on human participants in accordance with the local legislation and institutional requirements. Written informed consent for participation was not required for this study in accordance with the national legislation and the institutional requirements.

## Author Contributions

YL developed the theoretical framework and worked on literature review and manuscript writing. JL developed the theoretical framework and worked on data collection and analysis. Both authors contributed to the article and approved the submitted version.

## Conflict of Interest

The authors declare that the research was conducted in the absence of any commercial or financial relationships that could be construed as a potential conflict of interest.

## Publisher’s Note

All claims expressed in this article are solely those of the authors and do not necessarily represent those of their affiliated organizations, or those of the publisher, the editors and the reviewers. Any product that may be evaluated in this article, or claim that may be made by its manufacturer, is not guaranteed or endorsed by the publisher.

## References

[B1] Ajzen I. (2002). Perceived behavioral control, self-efficacy, locus of control, and the theory of planned behavior. *J. Appl. Soc. Psychol.* 32 665–683. 10.1111/j.1559-1816.2002.tb00236.x

[B2] Alalwan A. A. (2018). Investigating the impact of social media advertising features on customer purchase intention. *Int. J. Inf. Manag.* 42 65–77. 10.1016/j.ijinfomgt.2018.06.001

[B3] Al-Sabbahy H. Z. Ekinci Y. Riley M. (2004). An investigation of perceived value dimensions: implications for hospitality research. *J. Trav. Res.* 42 226–234. 10.1177/0047287503258841

[B4] Bang H. K. Ellinger A. E. Hadjimarcou J. Traichal P. A. (2000). Consumer concern, knowledge, belief, and attitude toward renewable energy: an application of the reasoned action theory. *Psychol. Mark.* 17 449–468. 10.1002/(SICI)1520-6793(200006)17:6<449::AID-MAR2>3.0.CO;2-8

[B5] Bianchi E. Bruno J. M. Sarabia-Sanchez F. J. (2019). The impact of perceived CSR on corporate reputation and purchase intention. *Eur. J. Manag. Bus. Econ.* 28 206–221. 10.1016/j.ridd.2012.05.006 22699256

[B6] Cai S. Xu Y. (2011). Designing not just for pleasure: effects of web site aesthetics on consumer shopping value. *Int. J. Electron. Comm.* 15 159–188. 10.2753/JEC1086-4415150405

[B7] Chen Y. S. Chang C. H. (2012). Enhance green purchase intentions: the roles of green perceived value, green perceived risk, and green trust. *Manag. Decis.* 50 502–520. 10.1108/00251741211216250

[B8] Chen Z. X. Aryee S. (2007). Delegation and employee work outcomes: an examination of the cultural context of mediating processes in China. *Acad. Manag. J.* 50 226–238. 10.5465/amj.2007.24162389

[B9] Chetioui Y. Benlafqih H. Lebdaoui H. (2020). How fashion influencers contribute to consumers’ purchase intention. *J. Fash. Mark. Manag.* 24 361–380. 10.1108/JFMM-08-2019-0157

[B10] Diefenbach S. Hassenzahl M. (2011). The dilemma of the hedonic-appreciated, but hard to justify. *Interact. Comput.* 23 461–472. 10.1016/j.intcom.2011.07.002

[B11] Douglass R. B. (1977). *Belief, Attitude, Intention, and Behavior: An Introduction to Theory and Research.* University Park, PA: Penn State University Press.

[B12] Du H. M. Zhang S. M. (2016). The cloud design system building research of tourist Souvenirs. *DEStech Trans. Eng. Technol. Res.* 8, 274–281.

[B13] Eggert A. Ulaga W. (2002). Customer perceived value: a substitute for satisfaction in business markets? *J. Bus. Industr. Mark.* 17 107–118. 10.1186/s13054-016-1208-6 27885969 PMC5493079

[B14] Erez M. Earley P. C. (1993). *Culture, Self-Identity, and Work.* Oxford: Oxford University Press on Demand. 10.1093/acprof:oso/9780195075809.001.0001

[B15] Farh J. L. Earley P. C. Lin S. C. (1997). Impetus for action: a cultural analysis of justice and organizational citizenship behavior in Chinese society. *Admin. Sci. Q.* 42 421–444. 10.2307/2393733

[B16] Farh J. L. Hackett R. D. Liang J. (2007). Individual-level cultural values as moderators of perceived organizational support-employee outcome relationships in China: comparing the effects of power distance and traditionality. *Acad. Manag. J.* 50 715–729. 10.5465/amj.2007.25530866

[B17] Gill H. Boies K. Finegan J. E. McNally J. (2005). Antecedents Of trust: establishing a boundary condition for the relation between propensity to trust and intention to trust. *J. Bus. Psychol.* 19 287–302. 10.1007/s10869-004-2229-8

[B18] Goodman L. A. (1961). Snowball sampling. *Ann. Math. Stat.* 32 148–170. 10.1214/aoms/1177705148

[B19] Gounaris S. (2005). Measuring service quality in b2b services: an evaluation of the SERVQUAL scale vis-à-vis the INDSERV scale. *J. Serv. Mark.* 19 421–435. 10.1108/08876040510620193

[B20] Hagtvedt H. Patrick V. M. (2014). Consumer response to overstyling: balancing aesthetics and functionality in product design. *Psychol. Mark.* 31 518–525. 10.1002/mar.20713

[B21] Hsiao K. L. (2013). Android smartphone adoption and intention to pay for mobile internet: perspectives from software, hardware, design, and value. *Lib. Hi Tech.* 31 216–235. 10.1108/07378831311329022

[B22] Hui C. Lee C. Rousseau D. M. (2004). Employment relationships in China: do workers relate to the organization or to people? *Organ. Sci.* 15 232–240. 10.1287/orsc.1030.0050 19642375

[B23] Hui C. Wong A. Tjosvold D. (2007). Turnover intention and performance in China: the role of positive affectivity, Chinese values, perceived organizational support and constructive controversy. *J. Occup. Organ. Psychol.* 80 735–751. 10.1348/096317906X171037

[B24] Jiang Q. Yao Y. Sun M. (2019). “On the development of cultural and creative industry in museums: a case study of Beijing palace museum,” in *Proceedings of the Fourth International Conference on Economic and Business Management (FEBM 2019)*, (Amsterdam: Atlantis Press), 286–291. 10.2991/febm-19.2019.67

[B25] Karnowski V. Leonhard L. Kümpel A. S. (2018). Why users share the news: a theory of reasoned action-based study on the antecedents of news-sharing behavior. *Commun. Res. Rep.* 35 91–100. 10.1080/08824096.2017.1379984

[B26] Kirkman B. L. Shapiro D. L. (2001). The impact of cultural values on job satisfaction and organizational commitment in self-managing work teams: the mediating role of employee resistance. *Acad. Manag. J.* 44 557–569. 10.5465/3069370 3069370

[B27] Kleijnen M. De Ruyter K. Wetzels M. (2007). An assessment of value creation in mobile service delivery and the moderating role of time consciousness. *J. Retail.* 83 33–46. 10.1016/j.jretai.2006.10.004

[B28] Ko E. Kim E. Y. Lee E. K. (2009). Modeling consumer adoption of mobile shopping for fashion products in Korea. *Psychol. Mark.* 26 669–687. 10.1002/mar.20294

[B29] Li J. Han X. Wang W. Sun G. Cheng Z. (2018). How social support influences university students’ academic achievement and emotional exhaustion: the mediating role of self-esteem. *Learn. Individ. Differ.* 61 120–126. 10.1016/j.lindif.2017.11.016

[B30] Li J. Yang F. Qi J. Sun R. Geng R. (2021). The influence of job satisfaction on entrepreneurial intention: a cross-level investigation. *Int. Small Bus. J. Res. Entrep.* 40 385–402. 10.1177/02662426211018831

[B31] Li Z. Shu S. Shao J. Booth E. Morrison A. M. (2021). Innovative or Not? The effects of consumer perceived value on purchase intentions for the palace museum,s cultural and creative products. *Sustainability* 13:2412. 10.3390/su13042412

[B32] Liu Y. (2003). Engineering aesthetics and aesthetic ergonomics: theoretical foundations and a dual-process research methodology. *Ergonomics* 46 1273–1292. 10.1080/00140130310001610829 14612319

[B33] Lu H. P. Hsiao K. L. (2010). The influence of extro/introversion on the intention to pay for social networking sites. *Inf. Manag.* 47 150–157. 10.1016/j.im.2010.01.003

[B34] McClure C. Seock Y. K. (2020). The role of involvement: investigating the effect of brand’s social media pages on consumer purchase intention. *J. Retail. Consum. Serv.* 53:101975. 10.1016/j.jretconser.2019.101975

[B35] Montano D. E. Kasprzyk D. (2015). Theory of reasoned action, theory of planned behavior, and the integrated behavioral model. *Health Behav. Theory Res. Pract.* 70:231.

[B36] Nechita F. (2014). The new concepts shaping the marketing communication strategies of museums. *Bull. Transilvania Univ. Braşov Ser. VII Soc. Sci. Law* 7 269–278.

[B37] Ng T. W. Sorensen K. L. Yim F. H. (2009). Does the job satisfaction—job performance relationship vary across cultures? *J. Cross Cult. Psychol.* 40 761–796. 10.1177/0022022109339208

[B38] Ngo H. Y. Li H. (2015). Chinese traditionality and career success: mediating roles of procedural justice and job insecurity. *Career Dev. Int*. 20 627–645. 10.1108/CDI-08-2014-0112

[B39] Norman D. A. (2004). *Emotional Design: Why We Love (or Hate) Everyday Things.* New York, NY: Basic Civitas Books.

[B40] Porter M. E. (1985). Creating and sustaining superior performance. *Compet. Adv.* 167 167–206.

[B41] Schultz L. (2005). *Effects of Graphical Elements on Perceived Usefulness of a Library.* Stephenville, TX: Tarleton State University.

[B42] Shahid Z. Hussain T. Zafar F. (2017). The impact of brand awareness on the consumers’ purchase intention. *J. Mark. Consum. Res.* 33 34–38. 10.4172/2168-9601.1000223

[B43] Sheth J. N. Newman B. I. Gross B. L. (1991). Why we buy what we buy: a theory of consumption values. *J. Bus. Res.* 22 159–170. 10.3389/fpsyg.2021.829696 35126270 PMC8811303

[B44] Shi A. Huo F. Hou G. (2021). Effects of design aesthetics on the perceived value of a product. *Front. Psychol.* 12:670800. 10.3389/fpsyg.2021.670800 34393904 PMC8359925

[B45] Snoj B. Korda A. P. Mumel D. (2004). The relationships among perceived quality, perceived risk and perceived product value. *J. Prod. Brand Manag.* 13 156–167. 10.1108/10610420410538050

[B46] Song Y. H. Li M. H. (2018). Research on cultural and creative product development based on museum resources. *IOP Conf. Ser. Mater. Sci. Eng*. 452:022090. 10.1088/1757-899X/452/2/022090

[B47] Spreitzer G. M. Perttula K. H. Xin K. (2005). Traditionality matters: an examination of the effectiveness of transformational leadership in the United States and Taiwan. *J. Organ. Behav.* 26 205–227. 10.1002/job.315

[B48] Stanton S. J. Townsend J. D. Kang W. (2016). Aesthetic responses to prototypicality and uniqueness of product design. *Mark. Lett.* 27 235–246. 10.1007/s11002-015-9368-8

[B49] Sweeney J. C. Soutar G. N. (2001). Consumer perceived value: the development of a multiple item scale. *J. Retail.* 77 203–220. 10.1016/S0022-4359(01)00041-0

[B50] Talke K. Salomo S. Wieringa J. E. Lutz A. (2009). What about design newness? Investigating the relevance of a neglected dimension of product innovativeness. *J. Prod. Innov. Manag.* 26 601–615. 10.1111/j.1540-5885.2009.00686.x

[B51] Toufani S. Stanton J. P. Chikweche T. (2017). The importance of aesthetics on customers’ intentions to purchase smartphones. *Mark. Intell. Plann.* 35 316–338. 10.1108/MIP-12-2015-0230

[B52] Wang L. Bishop J. W. Chen X. Scott K. D. (2002). Collectivist orientation as a predictor of affective organizational commitment: a study conducted in China. *Int. J. Organ. Anal.* 10 226–239. 10.1108/eb028951

[B53] Weng J. T. de Run E. C. (2013). Consumers’ personal values and sales promotion preferences effect on behavioural intention and purchase satisfaction for consumer product. *Asia Pac. J. Mark. Log.* 25 70–101. 10.1108/13555851311290948

[B54] Wood C. M. Scheer L. K. (1996). Incorporating perceived risk into models of consumer deal assessment and purchase intent. *ACR North Am. Adv.* 23 399–404.

[B55] Woodruff R. B. (1997). Customer value: the next source for competitive advantage. *Journal of the academy of marketing science* 25 139–153. 10.1007/BF02894350

[B56] Xu H. (2017). *The process of Forbidden City’s Internet celebrity.* Available online at: http://www.zxart.cn/News/194/104273.html (accessed February 20, 2021).

[B57] Yang K. S. Yu A. B. Yeh M. H. (1989). “Chinese individual modernity and traditionality: Construct definition and measurement,” in *Proceedings of the Interdisciplinary Conference on Chinese Psychology and Behavior*, Vol. 2870354. Taipei.

[B58] Yu X. Wang X. (2021). The effects of entrepreneurial bricolage and alternative resources on new venture capabilities: evidence from China. *J. Bus. Res.* 137 527–537. 10.1016/j.jbusres.2021.08.063

[B59] Yu X. Li Y. Su Z. Tao Y. Nguyen B. Xia F. (2020). Entrepreneurial bricolage and its effects on new venture growth and adaptiveness in an emerging economy. *Asia Pac. J. Manag.* 37 1141–1163. 10.1007/s10490-019-09657-1

[B60] Yuan Y. Lai F. Chu Z. (2019). Continuous usage intention of Internet banking: a commitment-trust model. *Inf. Syst. Bus. Manag.* 17 1–25. 10.1007/s10257-018-0372-4

[B61] Zeithaml V. A. (1988). Consumer perceptions of price, quality, and value: a means-end model and synthesis of evidence. *J. Mark.* 52 2–22. 10.1177/002224298805200302

[B62] Zhang A. Y. Song L. J. Tsui A. S. Fu P. P. (2014). Employee responses to employment-relationship practices: the role of psychological empowerment and traditionality. *J. Organ. Behav.* 35 809–830. 10.1002/job.1929

[B63] Zhao H. Liu W. Li J. Yu X. (2019). Leader-member exchange, organizational identification, and knowledge hiding: the moderating role of relative leader-member exchange. *J. Organ. Behav.* 40 834–848. 10.1002/job.2359

